# Novel analgesic ω-conotoxins from the vermivorous cone snail *Conus moncuri* provide new insights into the evolution of conopeptides

**DOI:** 10.1038/s41598-018-31245-4

**Published:** 2018-09-07

**Authors:** Silmara R. Sousa, Jeffrey R. McArthur, Andreas Brust, Rebecca F. Bhola, K. Johan Rosengren, Lotten Ragnarsson, Sebastien Dutertre, Paul F. Alewood, Macdonald J. Christie, David J. Adams, Irina Vetter, Richard J. Lewis

**Affiliations:** 1 IMB Centre for Pain Research, Institute for Molecular Bioscience, The University of Queensland, Brisbane, QLD 4072 Australia; 2 Illawarra Health and Medical Research Institute, University of Wollongong, Wollongong, NSW 2522 Australia; 3 School of Biomedical Sciences, Faculty of Medicine, The University of Queensland, Brisbane, QLD 4072 Australia; 4 Discipline of Pharmacology, The University of Sydney, Sydney, NSW 2006 Australia; 5 School of Pharmacy, The University of Queensland, Brisbane, QLD 4102 Australia; 6 Institut des Biomolécules Max Mousseron, UMR 5247, Université Montpellier - CNRS, Place Eugène Bataillon, 34095 Montpellier Cedex 5, France

## Abstract

Cone snails are a diverse group of predatory marine invertebrates that deploy remarkably complex venoms to rapidly paralyse worm, mollusc or fish prey. ω-Conotoxins are neurotoxic peptides from cone snail venoms that inhibit Ca_v_2.2 voltage-gated calcium channel, demonstrating potential for pain management via intrathecal (IT) administration. Here, we isolated and characterized two novel ω-conotoxins, MoVIA and MoVIB from *Conus moncuri*, the first to be identified in vermivorous (worm-hunting) cone snails. MoVIA and MoVIB potently inhibited human Ca_v_2.2 in fluorimetric assays and rat Ca_v_2.2 in patch clamp studies, and both potently displaced radiolabeled ω-conotoxin GVIA (^125^I-GVIA) from human SH-SY5Y cells and fish brain membranes (IC_50_ 2–9 pM). Intriguingly, an arginine at position 13 in MoVIA and MoVIB replaced the functionally critical tyrosine found in piscivorous ω-conotoxins. To investigate its role, we synthesized MoVIB-[R13Y] and MVIIA-[Y13R]. Interestingly, MVIIA-[Y13R] completely lost Ca_v_2.2 activity and MoVIB-[R13Y] had reduced activity, indicating that Arg at position 13 was preferred in these vermivorous ω-conotoxins whereas tyrosine 13 is preferred in piscivorous ω-conotoxins. MoVIB reversed pain behavior in a rat neuropathic pain model, confirming that vermivorous cone snails are a new source of analgesic ω-conotoxins. Given vermivorous cone snails are ancestral to piscivorous species, our findings support the repurposing of defensive venom peptides in the evolution of piscivorous *Conidae*.

## Introduction

Cone snails are a diverse group of predatory marine invertebrates that deploy remarkably complex venoms to rapidly paralyse worm, mollusc or fish prey^1^. Each venom contains >1000 peptides^1^ named conopeptides or conotoxins, with venom complexity correlating with dietary breadth^2^. Most characterised conotoxins target ion channels in the peripheral and central nervous systems and muscle cells, providing a rich source of potent and selective molecules with potential to treat a variety of diseases, including pain^3^. ω-Conotoxin are a class of conopeptides in the knottin family that potently inhibit the mammalian neuronal Ca_v_ channels, including Ca_v_2.2^3^. Ca_v_2.2 is highly expressed in the superficial layers of the spinal cord as well as dorsal root ganglion neurons where it plays an important role in pain signal processing^4^. Accordingly, ω-conotoxins that selectively inhibit Ca_v_2.2 produce anti-nociceptive (analgesic) effects when administered spinally in animals and humans^5–8^, including MVIIA (Prialt) from *Conus magus*, which is currently marketed for intrathecal use in the treatment of severe chronic pain^5,9^.

Most cone snails feed on polychaete worms, and reconstruction of their evolution supports the hypothesis that ancestral cone snails were vermivorous^10–15^. To-date, ω-conotoxins have only been reported in the venom of fish hunting cone snails, where they were initially proposed to contribute to the “motor cabal” of conotoxins evolved for predation^16^ but more recently they have been identified as playing major defensive role^17^. Given ω-conotoxins were hypothesized to be repurposed from a defensive role in ancestral worm hunting species that facilitated a shift to fish hunting^12^, we used a Ca_v_2.2 screen to identify two novel ω-conotoxins MoVIA and MoVIB in the venom of *Conus moncuri*, a western Pacific worm hunting (vermivorous) cone snail^18,19^. *C. moncuri* was initially considered a synonym of *Conus litteratus* (subgenus Elisaconus) but was recently recognized as a new species^20^ and placed in the Embrikena subgenus^21^. Interestingly, these new ω-conotoxins had an arginine at position 13 instead of a tyrosine previously shown to be crucial for ω-conotoxin activity^22,23^ and both preferentially targeted fish Ca_v_2.2, suggesting ω-conotoxins play a defensive role in vermivorous cone snail species^17^.

## Results

### Assay-guided Isolation, Sequencing Alignment and Chemical Synthesis

**Figure 1:**
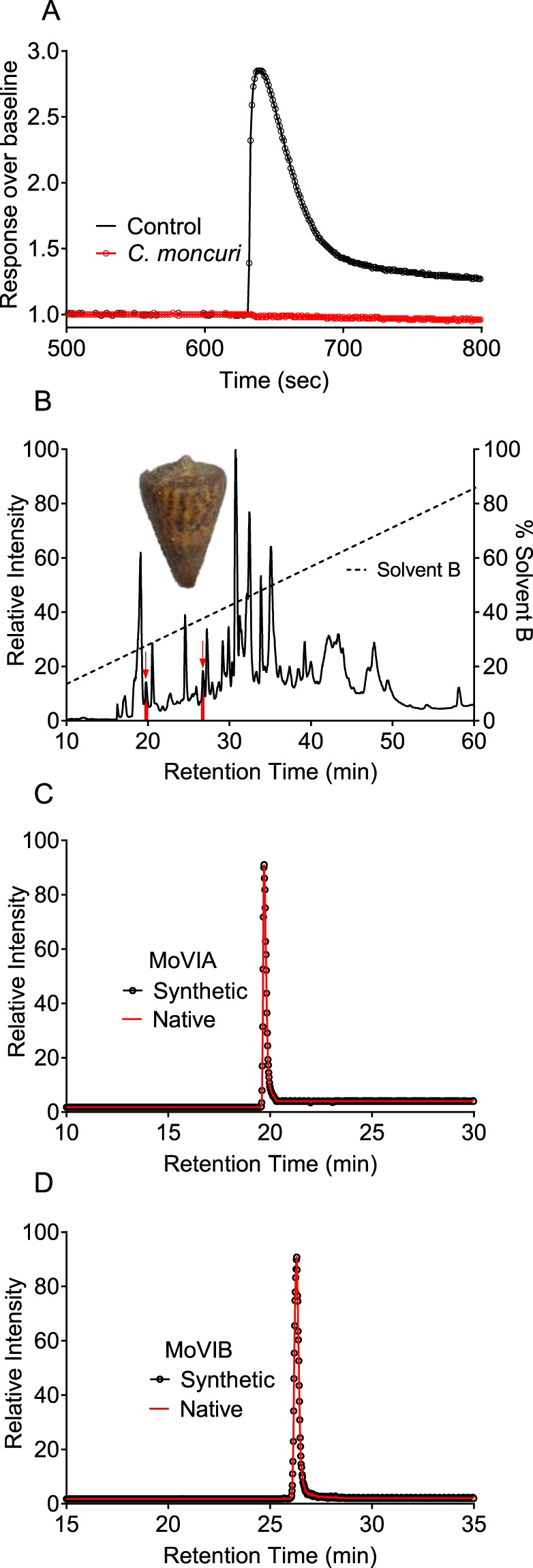
Assay-guided isolation of MoVIA–B from *C. moncuri* venom. (**A**) FLIPR calcium imaging assay. Crude venom from *C. moncuri* fully inhibited hCa_v_2.2 responses in SH-SY5Y cells. (**B**) Inset shows the shell of *C. moncuri* (image courtesy of Dr. Josh Wingerd) (**B**) Reversed-phase HPLC chromatography separated two peaks eluting at 19.5 and 26.5 min (indicated by red arrows), which inhibited hCa_v_2.2 activity in SH-SY5Y cells. (**C,D**) Extracted ion chromatograms of *C. moncuri* crude venom and synthetic peptides, confirming native MoVIA and MoVIB (red traces) coeluted with the synthetic forms (filled circles).

*C. moncuri* crude venom (~250 ng/µL) fully inhibited hCa_v_2.2 responses in SH-SY5Y cells (Fig. 1A). Two active fractions were isolated from *C. moncuri* crude venom (Fig. 1B) and sequenced, revealing two peptide sequences. Based on standard nomenclature, the peptides were named MoVIA (**C**KPOGSK**C**SOSMRD**CC**TT**C**ISYTKR**C**RKYYN) and MoVIB (**C**KPOGSK**C**SOSMRD**CC**TT**C**ISYTKR**C**RKYY). MoVIA and MoVIB are 31 and 30 amino acids in length, respectively, and contain a number of residues conserved in other ω-conotoxins, including lysine at position 2 (K2), glycine at position 5 (G5), hydroxyproline at position 10 (O), and arginine at position 25 (R25) (Table 1). Whereas most previously described ω-conotoxins have an amidated C-terminus^3^, mass/sequence calculation indicated MoVIA and MoVIB lacked a C-terminal modification. The mass and elution time of synthetic MoVIA (3605.49 Da and 19.5 min) and MoVIB (3492.42 Da and 26.5 min) were identical to the corresponding isolated native peptides (Fig. 1C,D) and these synthetic peptides were used for further studies.

**Table 1:** Related ω-conotoxin sequence alignments.

Conotoxin	Amino Acid Sequence^a^
MoVIA	**C**K**PO*GS***K**C*S*O*S*M**R*D***CC*TT***–**CI*S*Y*T***KR**C**RK**YY*N***
MoVIB	**C**K**PO*GS***K**C*S*O*S*M**R*D***CC*TT***–**CI*S*Y*T***KR**C**RK**YY**–
GVIA	**C**K***S*O*GSS*C*S*O*TS*Y*N*CC**R***S***–**C*N*OY*T***KR**C**–Y*– –
GVIIA	**C**K***S*O*GT*OC*S***R***G*M**R*D***CC*TS***–**CLLY*SN***K**C**RR**Y**– –
GVIIB	**C**K***S*O*GT*OC*S***R***G*M**R*D***CC*TS***–**CL*S*Y*SN***K**C**RR**Y**– –
MVIIA	**C**K***G***K***G*A**K**C*S***R**LMY***D*CC***TGS*C**R***SG***K–C*– – – – –

^a^Alignment generated manually with labeling scheme according to physicochemical criteria: Hydrophobic amino acids in bold (C, P, M, I, Y, L, A), acidic in italic (D), basic in underline (RK), hydroxyl + amide + Gly in bold/italic (G, S, T, N) and PTM in bold/underline (O for hydroxyproline). ^*^Amidated C-terminus.

### MoVIA Precursor Peptide Sequence

**Figure 2:**
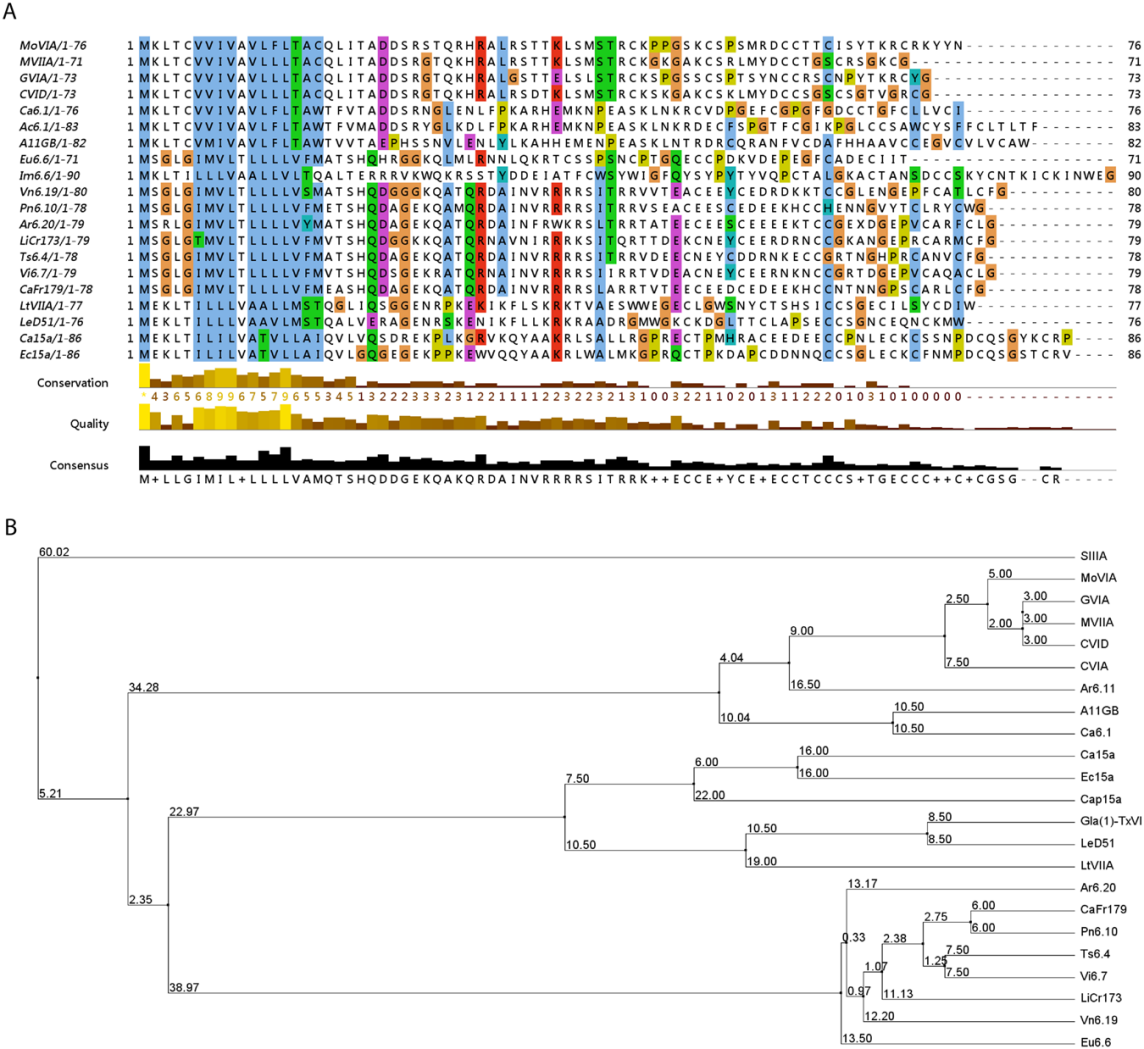
MoVIA precursor sequence and alignment. (**A**) Alignment of MoVIA precursor sequence with other conotoxins sequences belonging to the O1-superfamily. RACE PCR for cloning and amplification of the cDNA ends, using mRNA extracted from *C. moncuri* venom duct, identified a MoVIA nucleotide sequence of eighty-seven base pairs. The MoVIA precursor sequence was predicted through reverse translation using Expasy tools. (**B**) Cladogram tree generated in Jalview v2.8, with the tree roots show average distances. This cladogram reveals ω-MoVIA and ω-MoVIB are most closely related to ω-conotoxins from fish hunting cone snails.

RACE PCR on the venom duct of *C. moncuri* identified a cDNA clone encoding a new ω-conotoxin precursor. The MoVIA pre-propeptide sequence (MKLTCVVIVAVLFLTACQLITA*DDSRSTQRHRALRSTTKLSMSTR***CKPPGSKCSPSMRDCCTTCISYTKRCRKYYN**) comprising 76 amino acid residues, including a 22 amino acid signal sequence (underlined), a 23 amino acid pre-propeptide region (italicized), and a mature peptide region (bolded). The signal sequence was 100% identical to a precursor peptide from the vermivorous *C. pulicarius* and 95% identical to other precursor peptides from the O1 superfamily, including the vermivorous *C. betulinus* and the piscivorous MVIIA and GVIA. However, the mature peptide region was divergent from other ω-conotoxins, being only ~60% identical to GVIA, GVIIA and GVIIB, and 40% identical to MVIIA (see Fig. 2A, Table 1). Phylogenetic reconstruction of the untranslated and coding regions allowed the calculation of a cladogram tree (Fig. 2B), which shows MoVIA is most closely related to ω-conotoxins from piscivorous Conidae.

### 3D NMR Structure of MoVIB

**Figure 3:**
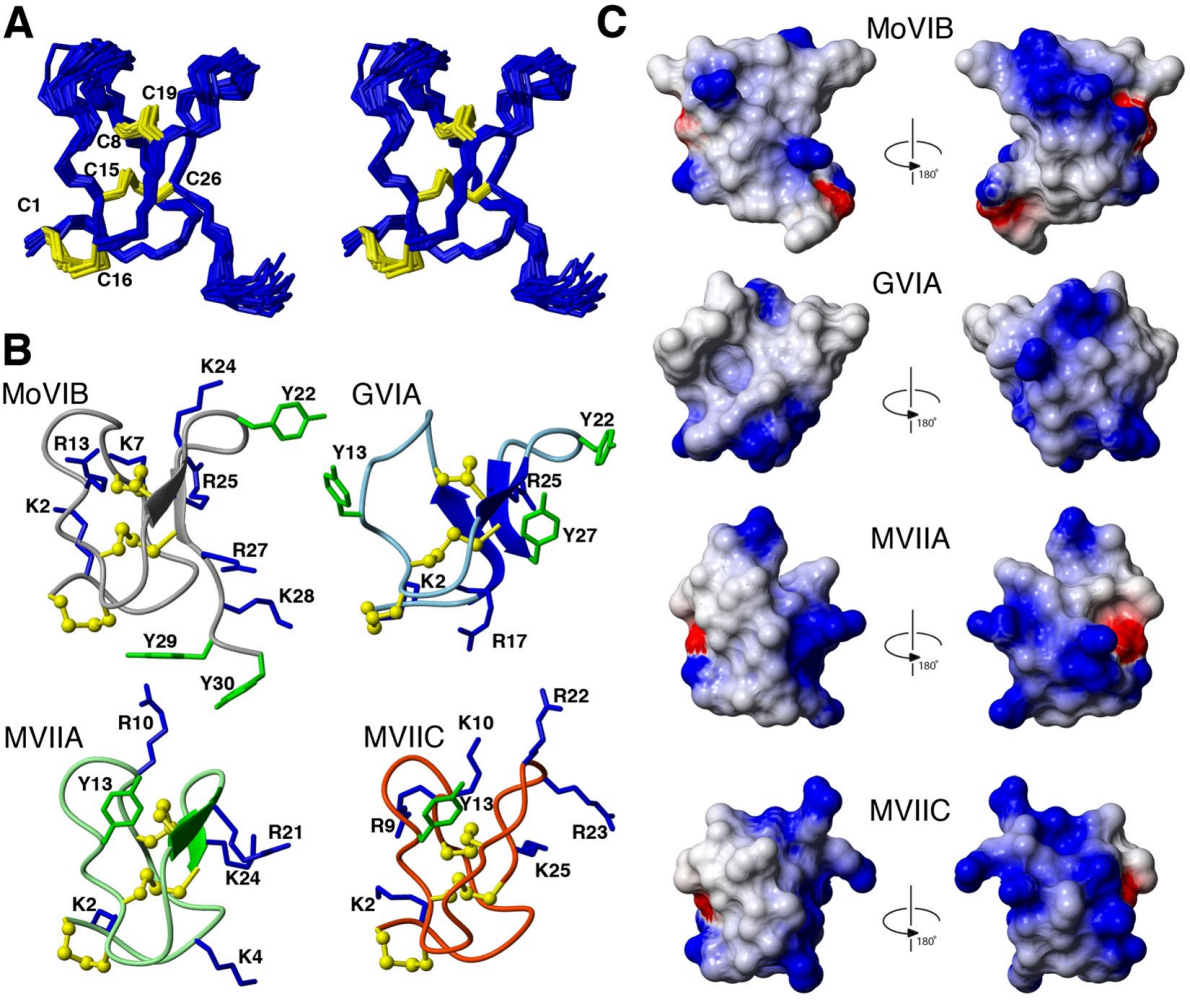
3D NMR solution structure of MoVIB and comparison with related ω-conotoxins structures. (**A**) Stereoview superposition of the family of 20 structures representing the MoVIB solution structure. (**B**) MoVIB backbone and side chains presented in a ribbon and ball-and-stick format, and compared to the GVIA, MVIIA and MVIIC structures. Highlighted are the residues identified as important for their pharmacophores, including Y13 (Tyr13) and K2 (Lys2). Disulfide bonds are shown in yellow (**C**) Electrostatic surfaces of MoVIB, GVIA, MVIIA and MVIIC. Positively charged residues are shown in blue and negatively charged residues in red.

In order to gain insight into the 3D structure, we subjected MoVIB to solution NMR spectroscopy studies at ultra-high field (900 MHz). The MoVIB NMR data were of high quality, with generally sharp line and distinct signal dispersion, consistent with a well-defined structure in solution. Collection of structural information from the data allowed calculation of the three-dimensional structure. From 50 refined structures the 20 best, judged on energies, consistency with the experimental data and covalent geometry (Fig. 3A, Table 2), were chosen to represent the MoVIB solution structure (PDB ID 6CEG; BMRB ID 30405).

**Table 2:** Energies and structural statistics for the family 20 structures of MoVIB with the best overall MolProbity score.

Parameters	Energies (kcal/mol)
Overall	−986.6 ± 31.6
Bonds	18.37 ± 1.40
Angles	56.18 ± 6.55
Improper	20.93 ± 2.44
Van der Waals	−104.3 ± 5.39
NOE	0.265 ± 0.019
cDih	0.179 ± 0.130
Dihedral	138.4 ± 1.75
Electrostatic	−1116.7 ± 33.8
**MolProbity Statistics**	
Clashes (>0.4 Å/1000 atoms)	8.28 ± 3.44
Poor rotamers	0.37 ± 1.14
Ramachandran Outliers (%)	0.19 ± 0.86
Ramachandran Favoured (%)	92.31 ± 1.25
**MolProbity score**	1.93 ± 0.14
MolProbity score percentile^a^	78.6 ± 6.86
Residues with bad bonds	1.43 ± 1.79
Residues with bad angles	0.00 ± 0.00
**Atomic RMSD (Å)**	
Mean global backbone (residues 1–28)	0.59 ± 0.19
Mean global heavy (residues 1–28)	1.26 ± 0.23
**Distance Restraints**	
Intraresidue (i-j = 0)	101
Sequential (/i-j/ = 1)	105
Medium range (/i-j/ < 5)	40
Long range (/i-j/>5)	97
Hydrogen bonds	6 (for 3 H-bonds)
Total	349
**Dihedral angle restraints**	
ϕ	9
Ψ	8
χ1	15
Total	32
**Violations from experimental restraints**	
Total NOE violations exceeding 0.3 Å	0 (highest 0.275)
Total Dihedral violations exceeding 3.0°	0 (highest 1.97)

The backbone structure of MoVIB (Fig. 3A) comprised a structural four-loop VI/VII framework cross-linked by disulfides to generate an ‘inhibitor cystine knot’ (ICK) motif reminiscent of other ω-conotoxins. The ICK, which features the three disulfides C1/C16, C8/C19, and C15/C26, is centered around a β-hairpin, which comprised residues 19–26 in MoVIB (Fig. 3A,B). The hydroxyl side chains from S6 and T17 form hydrogen bonds to the K7 and R27 backbone carbonyls, respectively. The two hydroxyprolines and one proline residue were all in a trans-conformation, based on ^13^C chemical shifts and sequential NOE patterns. Electrostatic mapping revealed that the MoVIB surface was highly charged (net charge +5.7) (Fig. 3C). The structures of GVIA, MVIIA and MVIIC (PDB ID code: GVIA, 1TTL; MVIIA, 1TTK; MVIIC, 1CNN), highlighting secondary structure, key pharmacophore residues and surface features, are shown for comparison (Fig. 3B,C).

### ^125^I-GVIA Binding Assays

**Figure 4:**
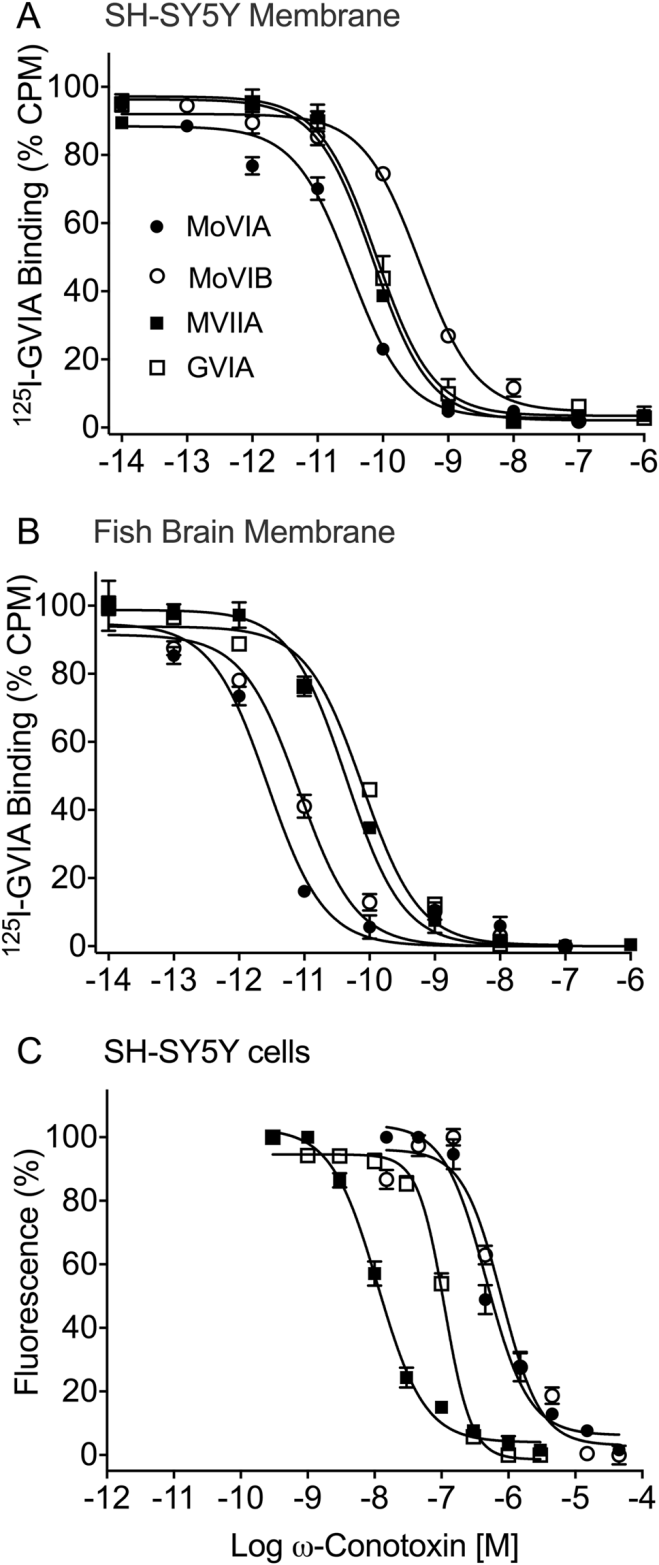
^125^I-GVIA Radioligand Binding and Functional calcium imaging assays. (**A**,**B**) MoVIA and MoVIB displaced ^125^I-GVIA from **(A)** SH-SY5Y cell and **(B)** fish brain membranes. (**C**) Concentration-response curves for ω-conotoxins MoVIA, MoVIB, GVIA and MVIIA. Data was calculated as mean ± SEM, n = 3–6 wells from 3–4 independent experiments. IC_50_s are given in Tables 3 and 4.

ω-Conotoxin GVIA from *C. geographus* is a high affinity Ca_v_2.2 ligand^24,25^ that can be radiolabeled for binding assays^26^. MoVIA and MoVIB fully displaced ^125^I-GVIA from SH-SY5Y cell membranes with similar potency (*p* < 0.001) to ω-conotoxins from piscivorous species (Fig. 4A,B, Table 3), suggesting they have overlapping binding site. Since MoVIA and MoVIB may have evolved for defense against fish predators, we determined if MoVIA and MoVIB could also displace ^125^I-GVIA from fish brain membranes. Consistent with this hypothesis, MoVIA and MoVIB displaced ^125^I-GVIA from fish brain with higher affinity than from human SH-SY5Y cell membranes (*p* < 0.05, two-way Anova) (Fig. 4, Table 3). Remarkably, MoVIA and MoVIB affinity for fish brain was significantly higher (*p* < 0.001) than ω-conotoxins from fish hunting species (Fig. 4B, Table 3).

**Table 3:** IC_50_ ± SEM (nM, N = 3) for displacement of ^125^I-GVIA binding.

ω-Ctx	Human SH-SY5Y Membrane	Fish brain Membrane
MoVIA	0.035 ± 0.017	0.004 ± 0.002
MoVIB	0.365 ± 0.204	0.006 ± 0.001
GVIA	0.078 ± 0.012	0.160 ± 0.086
MVIIA	0.068 ± 0.001	0.094 ± 0.042

### MoVIA and MoVIB Selectively Inhibit hCa_v_2.2 in Functional Assays

Consistent with results from the ^125^I-GVIA binding studies, MoVIA and MoVIB fully inhibited Ca_v_2.2 responses in fluorimetric calcium imaging assays in SH-SY5Y cells (Fig. 4C, Table 4). In contrast, both peptides were inactive (up to 30 µM) at endogenous human Ca_v_1.3 and Ca_v_3.1 (data not shown), indicating selectivity for hCa_v_2.

**Table 4:** IC_50_ ± SEM (μM) for ω-conotoxin inhibition of Ca_v_2.2.

ω-Conotoxin	SH-SY5Y fluorimetric hCa_v_2.2 assay (n = 3)	rDRG neuron N-type currents (n = 5)
MoVIA	0.33 ± 0.018	0.08 ± 0.01
MoVIB	0.60 ± 0.12	0.18 ± 0.03
MoVIB-[R13Y]	3.47 ± 0.55	0.90 ± 0.13
GVIA	0.17 ± 0.025	0.10^40^
MVIIA	0.024 ± 0.005	0.052^27^
MVIIA-[Y13R]	Inactive at 30 µM	ND

ND = Not determined.

**Figure 5:**
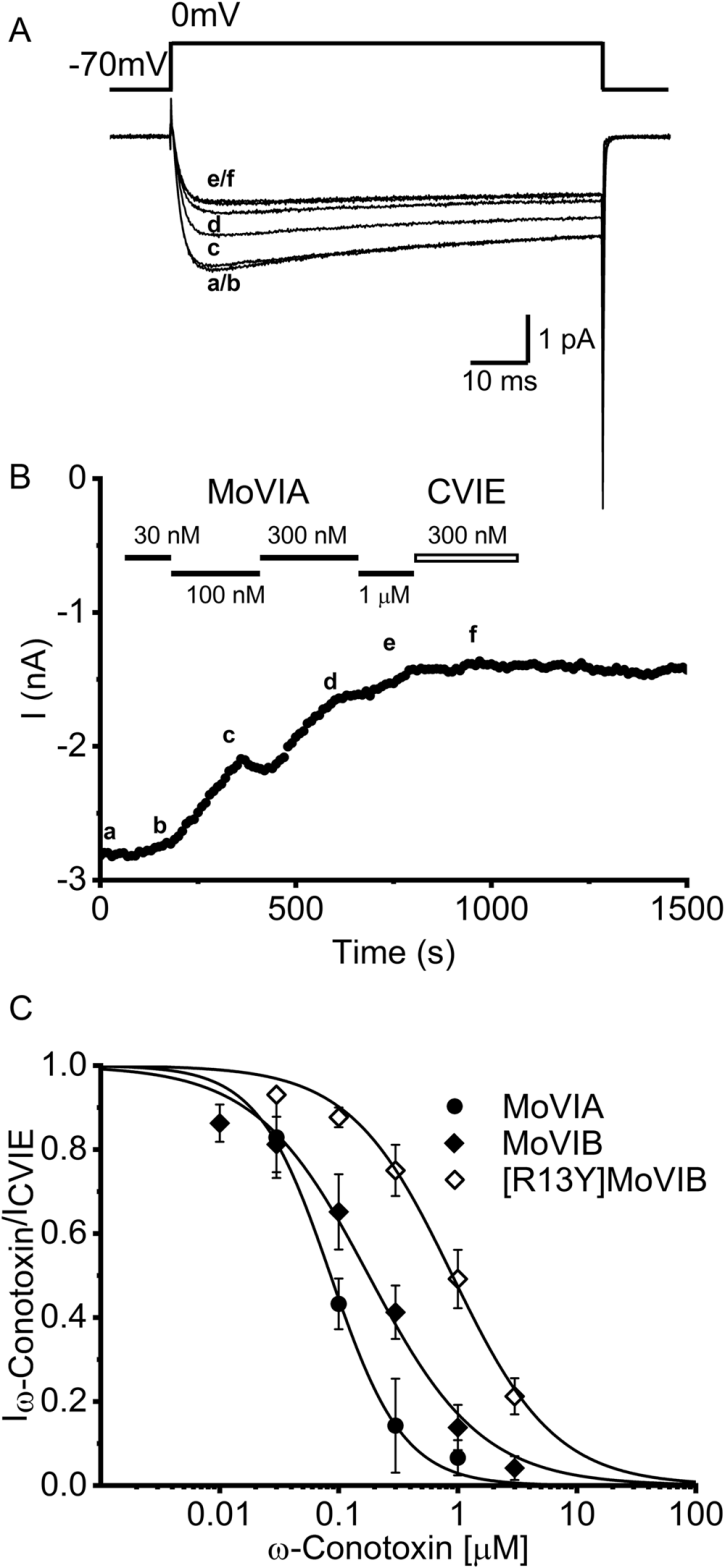
Inhibition of high voltage-activated (HVA) calcium channel currents in isolated rat DRG neurons. (**A**) Superimposed depolarization-activated whole-cell Ba^2+^ currents elicited by 75 ms voltage steps to 0 mV from a holding potential of −70 mV. (**B**) The current traces labelled correspond to time at which the Ba^2+^ current was recorded as indicated in (**B**). (**B**) Time course of experiment **(**in **A)**, with peak inward current amplitude plotted every 10 sec in the presence of increasing concentrations of MoVIA (30 nM–1 µM) followed by 300 nM CVIE. (**C**) Concentration-response relationships obtained for MoVIA, MoVIB and MoVIB-[R13Y]. Data points represent mean ± SEM of 5 independent experiments. IC_50_s are given in Table 4.

In rat dorsal root ganglia (rDRG) neurons, high voltage-activated (HVA) N-and P/Q-type calcium currents (Ca_v_2.2 and Ca_v_2.1 channels, respectively) contribute a major components of the endogenous calcium channel current^27,28^. In order to gain insight into the analgesic potential of MoVIA and MoVIB, we assessed their function on depolarization-activated Ba^2+^ currents in rat DRG neurons. MoVIA and MoVIB inhibited HVA currents in a concentration-dependent manner (Fig. 5A–C, Table 4). MoVIA inhibited ~34% of the whole-cell Ba^2+^ current that was not reversible following a 5-min washout, suggesting that these peptides have a slow off-rate. Further inhibition of N-type calcium channels with a high concentration of CVIE (300 nM)^29^ after MoVIA (3 µM) had no further effect on Ba^2+^ current amplitude (Fig. 5B), indicating N-type calcium channel currents were fully inhibited by MoVIA. A saturating concentration of the P/Q-type Ca_v_ inhibitor ω-agatoxin (10 µM) applied after MoVIA (3 µM) inhibited further 20% of the residual Ba^2+^ currents (data not shown), consistent with the percentage of P/Q-type current expressed in DRG neurons^27,29^, suggesting MoVIA had little effect at the P/Q-type currents endogenously expressed in rDRG.

### Role of Position 13 in ω-Conotoxins

**Figure 6:**
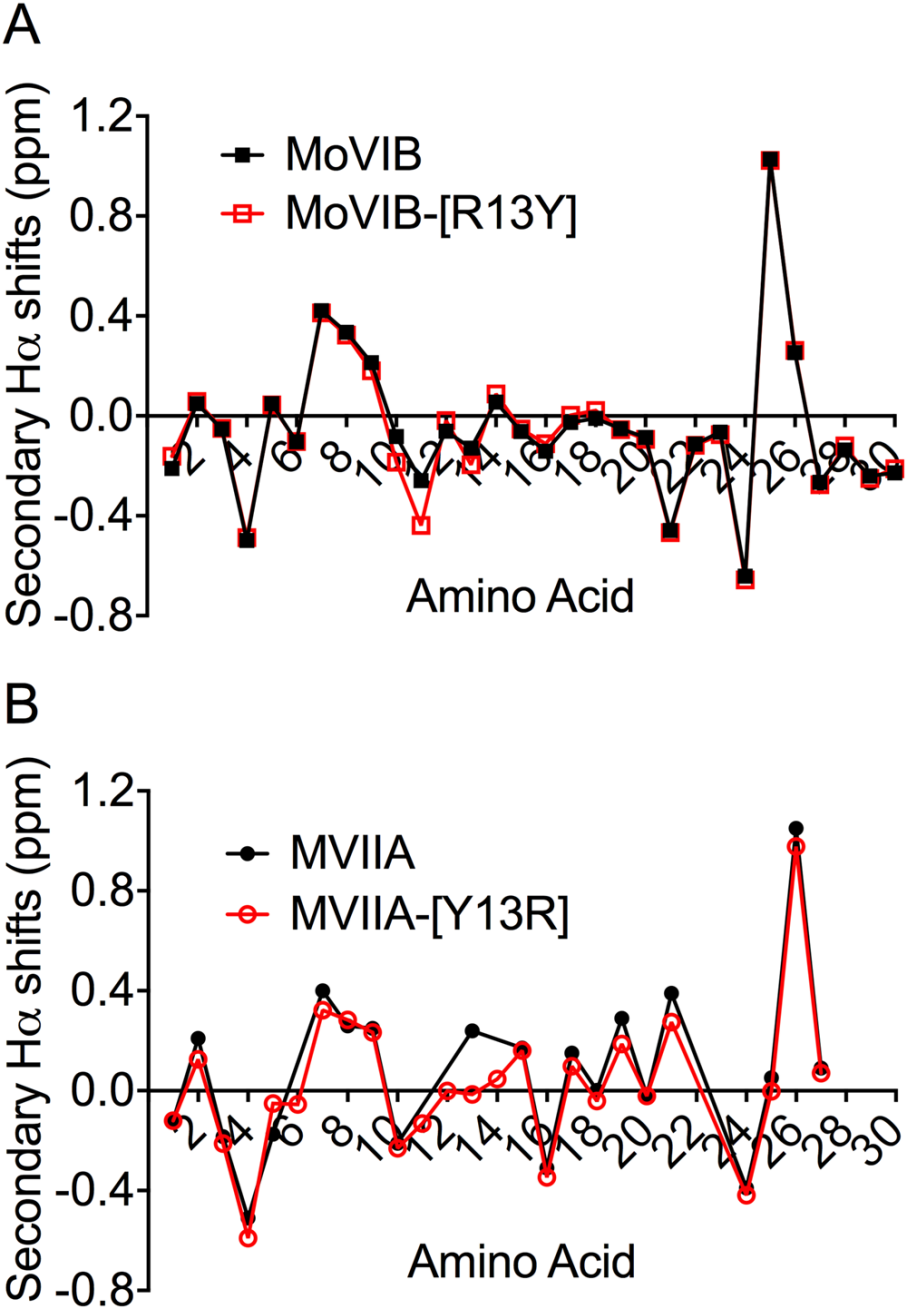
Hα secondary chemical shifts of MoVIB, MoVIB-[R13Y] and MVIIA-[Y13R]. The secondary Hα chemical shifts were determined by subtracting the shifts observed in random coil peptides from the shifts determined from the 2D NMR analysis. (**A**) Native MoVIB and the mutant MoVIB-[R13Y] (**B**) native MVIIA and the MVIIA-[Y13R]. Similar conformations for native and mutant peptides are evident from small deviations in chemical shifts, which are highly sensitive to structural changes.

Tyrosine at position 13 (Y13) has previously been shown to be essential for high affinity interactions of piscivorous ω-conotoxins at Ca_v_2.2^22,23,30,31^. In contrast, MoVIA and MoVIB maintain high Ca_v_2.2 affinity despite having an Arg (R13) instead of tyrosine at this key position. To investigate the structure-function at position 13, we synthesized and characterized MoVIB-[R13Y] and MVIIA-[Y13R]. NMR-derived secondary shifts of MoVIB-[R13Y] and MVIIA-[Y13R] were not significantly different from the corresponding native peptides (Fig. 6A,B), indicating that these replacements did not perturb the overall structure or fold.

In patch clamp assays using rat DRG, the potency of MoVIB-[R13Y] was ~5-fold lower compared to MoVIB (see Table 4). This result was supported by both fluorimetric calcium imaging and binding assays in SH-SY5Y cells, where the potency of MoVIB-[R13Y] was ~10-fold lower than wild type MoVIB (Table 4). In contrast, in calcium imaging assays using SH-SY5Y cells, MVIIA-[Y13R] was inactive at concentrations up to 30 µM (Table 4).

### Intrathecal MoVIB in a Rat Model of Neuropathic Pain

**Figure 7:**
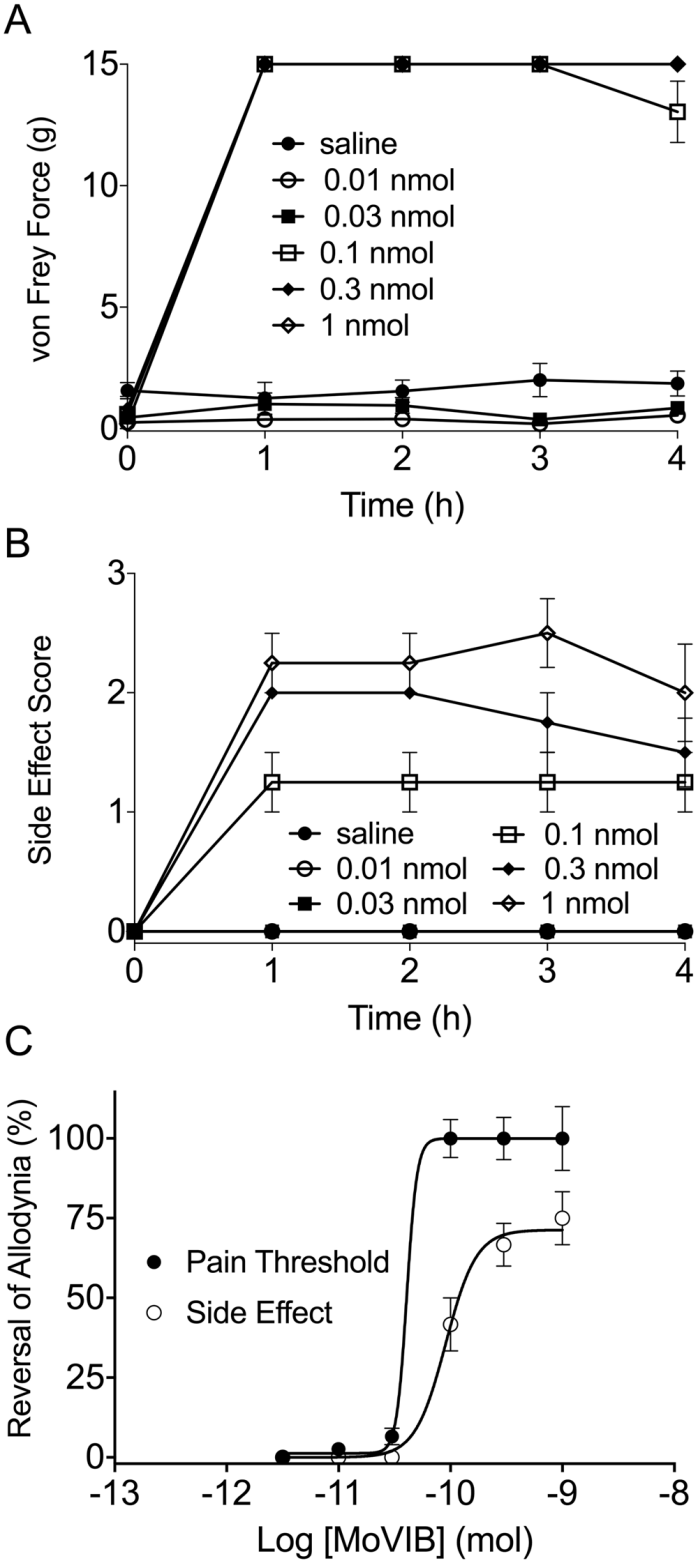
MoVIB effects on a rat partial nerve ligation model. (**A**) Intrathecal MoVIB significantly reversed mechanical allodynia in the PNL model (n = 3–6), in a dose dependent manner (ED_50_ 0.04 ± 0.01 nmol). Analgesic effect lasted for up to 4 h. (**B**) MoVIB side effects demonstrated using the rotarod. (**C**) Visual side effect score (0 = nil, 1 = mild, 2 = moderate, or 3 = severe) demonstrated that intrathecal MoVIB causes dose dependent side effects (ED_50_ 0.09 ± 0.05 nmol), with no significant improvement over 4 h (p > 0.05). (**D**) Dose response curves plotted for intrathecal MoVIB for calculation of the therapeutic index (TI) and safety window. ED_50_ data was calculated on PWT as 50% MPE and visual side effect score. Values denote mean ± SEM.

At days 10–11 post partial nerve ligation (PNL) surgery, rats developed a significant (p < 0.001) decrease in the PWT (paw withdrawal threshold; value of 0.6 ± 0.7 g, n = 24) compared to pre-surgery baseline (14.2 ± 1.6 g) (Fig. 7A), confirming the establishment of mechanical allodynia indicative of PNL-induced neuropathic pain. Intrathecal MoVIB dose-dependently reversed PWT (ED_50_ 0.04 ± 0.01 mol) associated with mechanical allodynia, at 0.1–1 nmol doses (n = 3–4, p < 0.01 one-way ANOVA) and this effect was irreversible over the first 4 h (p < 0.01–0.0001; two-way ANOVA) (Fig. 7A).

To quantify side effects of MoVIB we used a visual scoring method. MoVIB doses of 0.1–1 nmol produced effects on visual side effect scoring method that were significantly different from vehicle (p < 0.05; one way ANOVA) whereas the lower doses (0.01–0.03) produced no significant effect. The development of side effects was dose-dependent (ED_50_ 0.09 ± 0.05 nmol, Fig. 7B) and irreversible, lasting for up to 4 h (p < 0.0001; two-way ANOVA). The MoVIB therapeutic index, calculated as the ratio of the ED_50_s for the visual side effect score relative to mechanical PWT was 2.2, indicating a narrow safety window (Fig. 7C) though improved over the safety window for MVIIA (ED_50_ of 0.04 nmol and a TI of 0.7)^32^.

## Discussion

Cone snails have evolved different cabals comprising groups of venom peptides with complementary or synergistic pharmacology to facilitate prey capture^33–35^. Piscivorous cone snails use ω-conotoxins to inhibit vertebrate Ca_v_2.2 as part of a ‘motor cabal’ that blocks neuromuscular transmission and immobilizes fish^16,35^. Recently, ω-conotoxins were hypothesized to have evolved originally for defence by ancestral vermivorous species and later repurposed for fish hunting^12^. In the search for potential ancestral ω-conotoxins in worm-hunting species, we isolated two potent vertebrate-active ω-conotoxins from the venom of the vermivorous *Conus moncuri*^18^, suggesting that ω-conotoxins could have indeed evolved originally for defence in ancestral worm hunting species.

All venom peptides, including ω-conotoxins, are subjected to evolutionary pressures that enhance ancestral activities where they provide a competitive advantage^10–15^. Consistent with this view, similar evolutionary pressures likely also account for the highly potent ω-conotoxins found in vermivorous species. The close relatedness of vermivorous to piscivorous ω-conotoxin signal and propeptide sequences suggest they have related evolutionary origins and target related species. Since vermivorous cone snails are ancestral to piscivorous species^36^, this sequence relatedness confirms the potential for vertebrate-active ω-conotoxins in ancestral vermivorous to have evolved originally for defence against fish and to be later repurposed for fish hunting in piscivorous Conidae. However, MoVIA and MoVIB have an Arg13 replacing the functionally critical Tyr13 found in potent Ca_v_2.2 inhibitors from fish hunting species. Despite this important sequence difference, MoVIA and MoVIB potently displaced the prototypical ω-conotoxin GVIA from human SH-SY5Y cell membranes, indicating that vermivorous and piscivorous ω-conotoxins share an overlapping binding site. Interestingly, MoVIA and MoVIB displaced GVIA from fish brain membranes with even higher affinity and were more potent at fish calcium channels than GVIA and MVIIA from piscivorous Conidae. These data support MoVIA and MoVIB being positively selected to target Ca_v_2 in predatory fish as part of a defensive strategy, reminiscent of the δ-conotoxins found in the lightning-strike cabal of fish hunters that are also used defensively by other vermivorous cone snail species^37^.

In rat DRG neurons, N- and P/Q-type calcium channel currents are the predominant HVA calcium currents^28^. At saturating concentrations, MoVIA and MoVIB fully inhibited N-type (Ca_v_2.2) channels currents in rat DRG cells without significantly affecting P/Q-type (Ca_v_2.1) channels. Moreover, in human SH-SY5Y cells both MoVIA and MoVIB (30 µM) failed to inhibit Ca^2+^ influx associated with endogenously expressed Ca_v_1.3 and Ca_v_3.1. These findings position MoVIA and MoVIB as selective Ca_v_2.2 inhibitors, with similar pharmacological profile to the Ca_v_2.2-selective ω-conotoxins from piscivorous cone snails. Whereas the exact amino acid residues involved in selectivity remain incompletely defined, MoVIA and MoVIB contain a hydroxyproline at position 10 that is often found in Ca_v_2.2-selective peptides^38,39^. The reduced activity of MoVIA and MoVIB observed in Ca_v_2.2 functional *vs*. binding assays are well known for ω-conotoxins and have been attributed to the non-physiological conditions used to enhance ligand affinity in binding assays^31^. Alternatively, the presence of Ca_v_2.2 auxiliary subunits in the functional assays *vs*. their potential absence in binding assays may contribute to differences in potency, given ω-conotoxins have reduced affinity in the presence of the auxiliary α2δ subunit^24,29,40,41^.

To better understand the structure-function of these new vermivorous ω-conotoxins, we used NMR spectroscopy to determine the tertiary structure of MoVIB. Overall, the tertiary structure of MoVIB was similar to those of fish hunting ω-conotoxins, comprising six cysteines connected 1–4, 2–5, 3–6 to display four loops to match the VI/VII framework. MoVIB retained a number of residues in and around the ω-conotoxins pharmacophore^3^, including a significant number of positive (K2, K7, R13, K24, R25, R27 and K28) and hydrophobic (M12, I20, Y22,Y29, T30) residues. Comparing the NMR structures of MoVIB with those of other ω-conotoxins (Fig. 3) showed that Arg13 was oriented similarly to Tyr13 in MVIIA, GVIA and MVIIC, suggesting it may interact with a similar part of the pharmacophore perhaps also through hydrogen bonding interactions^22,30,31^.

Interestingly, MoVIA has an elongated C-terminus, with a Tyr30 and Asn31 in the last two positions, whereas the less potent MoVIB sequence finishes at Tyr30, indicating that Asn31 contributes to the higher affinity of MoVIA for Ca_v_2.2. Indeed, Asn31 is located near loop 4 that contributes to the ω-conotoxins pharmacophore^42^, with the most potent ω-conotoxins having an amidated C-termini^22,24^ and de-amidation reducing GVIA potency^31^. Therefore, it is conceivable that the side chain or backbone amide of Asn31 in MoVIA could mimic the contribution of the amidated C-terminus found in other ω-conotoxins. Despite Arg13 replacing the conserved Tyr13 critical for potent Ca_v_2.2 inhibition in piscivorous ω-conotoxins^22,23^, the pharmacology of MoVIA and MoVIB closely resembles that of ω-conotoxins from fish-hunting cone snails, including those from *C. consors*, *C. catus*, *C. fulmen*, *C. geographus*, *C. magus*, *C. radiatus*, *C. striatus* and *C. tulipa*^22,23,38,43,44^. Indeed, MoVIA and/or MoVIB may be ancestral to GVIIA and/or GVIIB, given Tyr13 is also replaced by Arg13 and both have similar elongated and non-amidated C-termini, albeit those from *C. geographus* are ~100-fold lower affinity than the highly homologous GVIA at mammalian Ca_v_2.2^16,45^. Similarly, the MVIIA-[Y13R] analogue also showed ~100-fold decrease in binding affinity over MVIIA and no functional activity at mammalian Ca_v_2.2. In contrast, the MoVIB-[R13Y] analogue failed to show enhanced binding affinity and potency to inhibit calcium influx in FLIPR assays and rat DRG neurons. Since NMR data indicates that MoVIB-[R13Y] and MoVIB have similar structures, it appears that Arg13 may be uniquely preferred to Tyr13 in vermivorous ω-conotoxins.

Consistent with Ca_v_2.2 being a validated analgesic target, rat behavioural studies using the PNL model of neuropathic pain confirmed the MoVIB analgesic activity. Intrathecal injection of MoVIB accompanied side effects common to ω-conotoxins, including shaking, tail twitching and serpentine tail movement, indicating that MoVIB would likely need to be carefully titrated intrathecally in a clinical setting to manage dose-limiting side effects also seen for ω-conotoxins MVIIA and CVID^46^. Nonetheless, we calculated an apparently improved safety window for MoVIB compared to that published for MVIIA^32^, which is currently marketed. Future studies will investigate the activity and the therapeutic index of MoVIA and MoVIB in other clinically relevant models of pain.

In conclusion, we have discovered and pharmacologically characterized two novel ω-conotoxins from the venom of a vermivorous cone snail, *Conus moncuri*. ω-MoVIA and ω-MoVIB had highest affinity for fish Ca_v_s, suggesting they were positively selected for defense against predation in this worm-hunting species. Given vermivorous cone snails are ancestral to piscivorous species, this finding supports the repurposing of defensive venom peptides during the evolution of piscivorous *Conidae*, as proposed previously^17^. Alternatively, MoVIA and MoVIB may be an example of convergent evolution of distantly related cone snail toxins that target similar pharmacology in different organisms^47,48^, although the striking structural and sequence similarities suggest otherwise. Like two modestly potent piscivorous ω-conotoxins GVIIA and GVIIB, these vermivorous ω-conotoxins possess an Arg13 instead of the otherwise critical Tyr13, providing new insight into the structural features required for high-affinity interactions of ω-conotoxins at Ca_v_2.2 channels.

## Methods

### Drugs and Chemicals

All drugs and chemicals were analytical reagent grade sourced from Sigma Aldrich, NSW, Australia, unless otherwise detailed throughout the text (in parentheses).

### Venom Extraction

Crude venom was stored at 4 °C for immediate use, or at −20 °C for long-term storage. Prior to fractionation crude venom was re-suspended in 30% acetonitrile/water (ACN/H_2_O; Sigma Aldrich, NSW, Australia), acidified with 0.1% trifluoroacetic acid (TFA) and centrifuged at 10,000 x g for 5 min. Soluble material was lyophilized and re-dissolved in 5% ACN/H_2_O. Protein content was estimated by measuring the absorbance at 280 nm (E280) using a Nanodrop (Thermo Scientific).

### Assay-Guided Isolation of MoVIA and MoVIB

Crude venoms were screened for Ca_v_2.2 activity using the human SH-SY5Y neuroblastoma cell line and the FLIPR^TETRA^ (Fluorescent Imaging Plate Reader, Molecular Devices, CA, USA) calcium imaging assay, as described previously^40^. Two Ca_v_2.2 inhibitors were isolated using Reversed Phase High Performance Liquid Chromatography (RP-HPLC) on a Vydac 218TP C18 (250 × 4.6 mm, 5 μm) analytical column. Fractions eluted at 0.7 ml/min with a linear gradient of 0–100% solvent B over 60 min (solvent A: 90% water, 0.1% formic acid; solvent B, 90% acetonitrile, 10% water, 0.09% formic acid) on a Dionex UltiMate® 3000 LCi solvent delivery system (Thermo Scientific). Fractions were collected on a Gilson FC204 (Gilson) fraction collector and lyophilized. Dried fractions were re-dissolved in physiological salt solution (PSS) assay buffer (composition in mM: NaCl 140, Glucose 11.5, KCl 5.9, MgCl_2_ 1.4, NaH_2_PO_4_ 1.2, NaHCO_3_ 5, CaCl_2_ 1.8, HEPES 10) and tested for Ca_v_2.2 channel activity in SH-SY5Y cells using the FLIPR platform.

Active Ca_v_2.2 inhibitor fractions were analyzed for purity by liquid chromatography/mass spectrometry (LC/MS) and their molecular weights determined using a matrix-assisted laser desorption/ionization time-of-flight (MALDI-TOF) mass spectrometry system (4700 Proteomics Analyzer, Applied Biosystems, Mulgrave, Australia), with an α-cyano-4-hydroxycinnamic acid matrix (CHCA [5 mg/mL]; Sigma Aldrich).

### Peptide Sequencing

We obtained the MoVIA and MoVIB N-terminal sequences using automated Edman degradation (Australian Proteome Analysis Facility) on a 494 Procise Protein Sequencing System (Applied Biosystems). The MoVIA and MoVIB samples were dissolved in urea (4 M) and ammonium bicarbonate (50 mM) and reduced using dithiothreitol (100 mM) at 56 °C for 1 h under argon. Then alkylated with acrylamide (220 mM) for 30 min in the dark, and reaction quenched by the addition of an excess of dithiothreitol. The sample mixtures were desalted by reverse RP-HPLC and collected fractions dried using a SpeedVac concentrator (Thermo Scientific). Subsequently, samples were loaded onto pre-cycled bioprene discs and subjected to 35 cycles of Edman degradation for N-terminal sequencing.

### RNA Extraction and RACE PCR

*C. moncuri* venom duct (1 mg) was carefully removed on ice, immediately placed in RNAlater® (Ambion, NSW, Australia) and kept at 4 °C. On the day of RNA extraction, the tissue was grounded and homogenized. Total RNA extraction was carried out using TRIZOL Reagent kit (Invitrogen, CA, USA), accordingly to the manufactures’ instructions. Isolated RNA was subsequently treated with RNase/DNase-free kit (Qiagen, Hilden, Germany) to remove any genomic DNA contamination. RNA concentration was determined by absorbance measurements at 260 nm. RNA purity/integrity was assessed by analyzing the ratio 260/280 nm using a NanoDrop (Thermo Scientific, MA, USA).

The resulting cDNA was used as template in a polymerase chain reaction (PCR). Primers used on RACE PCR were designed based on previously published sequences from members of the O-superfamily of vermivorous Conidae^49^. The 3′ RACE first strand cDNA was synthesized from 1 μg total RNA using FirstChoice RLM-RACE kit (Ambion), following the manufacturer’s instructions. Primer sequences were F1 = 5′-CATCGTCAAGATGAAACTGACGTG-3′ and R1 = 5′-CACAGGTATGGATGACTCAGG-3′. PCR reaction with 500 ng of cDNA as template was performed using FastStart Taq DNA polymerase (Roche, Basel, Switzerland), under the following cycling conditions: 95 °C for 4 min, followed by 40 cycles of 95 °C for 30 s, 58 °C for 30 s, and 72 °C for 1 min and a final elongation step at 72 °C for 7 min. PCR products were analysed and purified after separation on a 1% agarose gel, using a QIAquick Gel Extraction kit (Qiagen). Gel extracted PCR products were sequenced at the Australian Genome Research Facility (AGRF), using the forward and reverse primers F1 and R1, respectively. Sequencing data was transferred to Expasy Tools^50^ for sequence translation and amino acid sequence prediction. Sequences from other conotoxins belonging to the O-superfamily were retrieved either from GenBank^51^ or Conoserver^52^ and compared with MoVIA. Sequence alignment was performed using Clustal W^53^ and Jalview version 2.8^54^.

### Chemical Synthesis of MoVIA and MoVIB

Synthesis of MoVIA, MoVIB and analogues were performed using *in situ* neutralization Boc-SPPS on a Boc-Asn-PAM or Boc-Tyr-PAM resin employing HBTU/DIEA activation, respectively, as described previously^55^. Assembled peptidyl-resin was cleaved with hydrogen fluoride for 1 h using *p*-cresol/*p*-thio-cresol scavenger (10%) and crude peptide precipitated from ether, filtered and lyophilized from acetonitrile/H_2_O. After HPLC clean up, pure reduced peptides (20 mg each/[0.2 mg/mL]) were oxidized at pH 7.8 in a solution of 0.3 M NH_4_OAc/0.3 M guanidine-HCl in the presence of GSH/GSSG (100:10 mol eq). Two major peptide isomers were obtained after RP-HPLC in quantities of 1–2 mg, corresponding to MoVIA and MoVIB.

### Mass Spectrometry

LC-ESI-MS/MS was performed on synthetic MoVIA and MoVIB samples separated on a ZORBAX 300SB-C18 (2.1 × 100 mm × 1.8 µm) column, eluted with a Shimadzu 30 series HPLC system at 400 μl/min, with a linear gradient from 1–80% over 25 min. The eluent was analysed on a tripleTOF 5600 mass spectrometer (ABSCIEX, MA, USA) with a quadruple TOF system and a DuoSpray ionisation system. The ion-spray voltage was set to 5300 V, with full scanning over 250 ms, followed by full scan product ion data obtained in the information dependant acquisition (IDA) mode over 20 × 50 ms. The mass range was set to 300–4000 (*m/z*) for TOF MS mode and 80–4000 (*m/z*) for full scan TOF MS/MS mode. Buffer A was 0.1% FA and buffer B was 90% acetonitrile/0.1% FA. All data analysis was performed using Analyst 1.6 (ABSCIEX).

### 2D NMR Spectroscopy and 3D Structure Calculations

MoVIB and analogue peptides samples were prepared at 2 mg/ml in 90% H_2_O/10% D_2_O or 100% D_2_O (pH 5.0) for Nuclear Magnetic Resonance (NMR) spectroscopy studies. Two dimensional (2D) homonuclear ^1^H-^1^H total correlation spectroscopy (TOCSY**)**, nuclear Overhauser effect (NOESY) and exclusive correlation spectroscopy (ECOSY) datasets, and a 2D heteronuclear ^1^H-^13^C HSQC were recorded at 900 MHz on a Bruker Avance II spectrometer, equipped with a cryogenically cooled probe and processed using Topsin 3.0 (Bruker). Homonuclear data were recorded with 2048 data points in the direct dimension and 512 increments in the indirect dimension over a sweep-width of 12 ppm. The HSQC spectrum was recorded with an indirect dimension sweep-width of 106 ppm. Data analysis were performed using the Computer Aided Resonance Assignment (CARA) software^56^. Structural restraints derived from the NMR data included (i) Inter-proton distances derived from NOESY cross-peak intensities in spectra recorded in either H_2_O or D_2_O with a mixing time of 100 ms. (ii) Backbone dihedral angles (Phi and Psi) derived from a TALOS+^57,58^ analysis of Cα, Cβ, Hα and HN chemical shifts. (iii) Side chain dihedral angles (χ1) derived from analysis of ^3^J_HαHβ_ coupling constants and intra residual NOE patterns (iv) Hydrogen-bond restraints derived from amide exchange rates and analysis of preliminary structures. Nuclear Overhauser effect (NOE) cross peaks were manually picked and subsequently calibrated and assigned automatically using the automatic assignment and structure calculation module of CYANA 3.0^59^. For the final structures distance restraint lists from CYANA were used as input for simulated annealing and water minimization within CNS^60^, using protocols from the RECOORD database^61^, modified as described previously^62^. In the final round 50 structures were calculated, and the best 20 based on energies and quality of packing and geometry as judged by MOLPROBITY^63^ scores were chosen as representative of the solution structure of MoVIB. Figures were prepared using MOLMOL^64^.

### Fluorimetric calcium imaging assays

For assay-guided fractionation and Ca_v_ selectivity characterization of MoVIA, MoVIB and mutant peptides, we used a cell-based calcium-imaging fluorimetric assay and the FLIPR platform. Human neuroblastoma SH-SY5Y cells were exposed to saturating concentrations of the Ca_v_1 inhibitor nifedipine (10 µM) to isolate endogenous Ca_v_2.2^40^. For selectivity studies, we isolated Ca_v_1.3 using saturating concentrations of Ca_v_2.2 inhibitor CVID (3 µM). The small response remaining resistant was inhibited with saturating concentrations of the Ca_v_3 inhibitor mibefradil (30 µM)^40^. Briefly, we incubated SH-SY5Y cells with the Ca^2+^ dye Fluo 4 in the presence of Ca_v_ inhibitors for 30 min. Test toxins or controls were then added to the cells and responses monitored for 10 min. KCl stimulation buffer was then added to stimulate Ca_v_ channel opening and responses were recorded for an additional 5 min.

### Electrophysiological Recording from rat DRG neurons

Rat DRG neurons were enzymatically dissociated from 10–16 day old Wistar rats, as described previously^65^. We performed whole-cell patch clamp recording using a MultiClamp 700B Amplifier (Molecular Devices). Data was digitalized with a Digidata 1322 A (Molecular Devices), filtered at 10 kHz and sampled at 100 kHz using pClamp 9.2 software. External recording solution contained (in mM): 150 TEA-Cl, 2 BaCl_2_, 10 D-Glucose and 10 HEPES, adjusted to pH 7.4 with TEA-OH. The pipette electrodes had a final resistance of (1–3 MΩ), with an intracellular solution containing (mM): 140 CsCl, 1 MgCl_2_, 5 BAPTA and 10 HEPES adjusted to pH 7.2 with CsOH. High voltage-activated (HVA) calcium channel currents were recorded using Ba^2+^ as the charge carrier and by measuring peak inward current amplitude elicited by 75 ms voltage steps to 0 mV from a holding potential of −70 mV. After current achieved steady state, we applied toxins to the physiological solution and plotted the peak inward current amplitude every 10 s. Series resistance were typically compensated at 70–80%, and leak and capacitance currents were subtracted using a −P/4 pulse protocol. We used selective Ca_v_2.1 (P/Q-type) channel inhibitor Agatoxin-IVA (Abcam, Cambridge, United Kingdom) and CVIE, a selective Ca_v_2.2 (N-type) channel inhibitor, to examine the selectivity profile of MoVIA and MoVIB in rDRG.

### Radioligand Binding Assays

The cell membranes from SH-SY5Y and fish brain preparations and the radioligand binding assays using ω-peptide GVIA radiolabelled at Tyr^22^ ([^125^I]-GVIA, Perkin Elmer) were performed as described previously^26,40^.

### Partial Nerve Ligation-Induced Neuropathy in Rats

Animal experimentation followed the National Health and Medical Research Council Code of Practice for the Care and Use of Animals for Research in Australia, with the approval of the Royal North Shore Hospital Animal Care and Ethics Committee. Old male Sprague-Dawley rats (8–10 week, weighting 200–260 g) were housed three per enclosure and maintained on a standard 12 h light/dark cycle, with free access to food and water.

Tight partial nerve ligation of the sciatic nerve was carried out under isoflurane anaesthesia (1–3% in O_2_), as previously described^32^. To determine the establishment of neuropathy, PWTs were monitored using von Frey hair filaments and the up-down paradigm^66^. Briefly, a blinded experimenter applied six times von Frey hair stimulus of equal intensity (range 0.4–15 g) to each rat hind paw (at intervals of several seconds from time 0–4 h) and recorded the responses to stimuli (flinching and licking of the hind paws) and side effects. The average of these values per animal served as the pain related score, with no response to 15 g von Frey hair indicating the maximum possible effect (MPE). A visual score of 0–3, following previously described method^32,46,67^, was used to determine the degree of side effect.

One week after PNL surgery rats that developed significant neuropathy received intrathecal long-term polyethylene lumbar catheters inserted between vertebrae L5 and L6. von Frey measurements were taken before to set baseline and after intrathecal treatment with vehicle or MoVIB. MoVIB (0.01–1 nmol, n = 3–6 animals/dose) was freshly dissolved in 0.9% saline and injected via the catheter in a volume of 10 µl, followed by 15 µl of 0.9% saline to wash the drug from the catheter dead space. Control animals received 0.9% saline injections of equal volume. Following intrathecal injections, we monitored rat behaviour at every hour over a 4-h period. After each experiment, we checked the correct placement of catheters by injecting lignocaine (2%) and observing rapid bilateral hind limb paralysis. Animals that had no paralysis or lost weight after the catheter surgery were excluded from the analysis.

### Statistical analysis

Sigmoidal concentration-response curves and IC_50_ values were calculated using GraphPad Prism v5.0, following a nonlinear regression analysis with a four parameter (variable Hill slope) equation fitted to the functional data and a three parameter (Hill slope of −1) equation fitted to radioligand binding data. To calculate average peak current values we used Microsoft Excel version 12.2.0. All results were expressed as the mean ± standard error of the mean (SEM) determined from triplicate data from at least 3 independent experiments. Statistical significance was determined using analysis of variance (ANOVA), with statistical significance defined as p < 0.05, unless otherwise stated. We calculated the MPE values, from animal experiments applying the maximum pain threshold and maximum side effect score. ED_50_ values were calculated for % MPE data using a four parameter Hill slope with a variable slope, and compared using one-way or two-way ANOVA. When ANOVA tests were significant, we made post-hoc comparisons between drug/treatment groups and vehicle at individual time points using Bonferroni or Turkey adjustment for multiple comparisons.

## Data Availability

All data generated or analysed during this study are included in this published article.
